# Online Consumer Satisfaction During COVID-19: Perspective of a Developing Country

**DOI:** 10.3389/fpsyg.2021.751854

**Published:** 2021-10-01

**Authors:** Yonghui Rao, Aysha Saleem, Wizra Saeed, Junaid Ul Haq

**Affiliations:** ^1^Antai College of Economics & Management, Shanghai Jiao Tong University, Shanghai, China; ^2^School of Management, Zhejiang Shuren University, Hangzhou, China; ^3^Faculty of Management Sciences, Riphah International University, Faisalabad Campus, Punjab, Pakistan; ^4^Department of Professional Psychology, Bahria University, Islamabad, Pakistan

**Keywords:** consumer perception, online shopping, actual experiences, customer satisfaction, direct shopping, perceived risk, delight, outrage

## Abstract

A conceptual model based on the antecedents and consequences of online consumer satisfaction has been proposed and empirically proved in this study. Data were collected during Smart Lockdown of COVID-19 from 800 respondents to observe the difference between perceived and actual, and direct and indirect e-stores. Confirmatory factor analysis was used to observe the validity of the data set. The structural equation modeling technique was used to test the hypotheses. The findings indicated that consumers feel more satisfied when they shop through direct e-store than indirect e-store, whereas their perception and actual experience are different. Implications have also been added to the study.

## Introduction

Online shopping is the act of buying a product or service through any e-stores with the help of any website or app. Tarhini et al. (2021) stated that shopping through online channels is actively progressing due to the opportunity to save time and effort. Furthermore, online shopping varies from direct e-store and indirect e-store about their perception against the actual experience. Developing countries still face various conflicts and issues while promoting and utilizing e-commerce to the maximum compared with the developed countries (Rossolov et al., 2021). In the developing countries, the difference between the perception and actual experience of the consumers varies when buying from indirect e-store compared to the direct e-store. On the contrary, as the world has been suffering from the COVID-19 pandemic, it has brought drastic changes globally in many sectors, business being one of them. De Vos (2020) stated that a large-scale lockdown was imposed worldwide to prevent the virus from spreading.

To survive, switching traditional shopping or trade toward digital was one factor that captured the attention across the globe on a larger scale. In April 2020, Walmart reported a 74% increase in online sales even though they faced a low customer walk-in at stores (Nassauer, 2020; Redman, 2020). This upsurge of swift adoption of online channels has led this research to ask a few questions. First, what will be the difference between the perceived and the actual product purchased online? A recent study has documented that consumers bear actual risk after shopping through online channels (Yang et al., 2020). Research reported that 30% of the products through online channels get returned and are not according to their perception (Saleh, 2016). The same author also showed that the return and complaint rates are getting higher when consumers shop through an online channel.

Second, is there any difference between the perceived and the actual product purchase online from a direct e-store or an indirect e-store? Direct e-store means the online brand store, for example, Walmart, and indirect e-store means third-party stores such as Amazon, Alibaba, Jingdong (JD), and Daraz. The direct e-store strives hard to maintain a clear, potent perception in the mind of its buyer (Grewal et al., 2009). Kumar and Kim (2014) stated that a brand strengthening its relationship with its consumer satisfies its needs through the actual product or services. In the literature (Olotewo, 2017; Rossolov et al., 2021), it is stated that the shopping patterns of buyers from direct and indirect e-stores vary greatly, especially in the developing countries. In this way, when shopping through a direct e-store, consumers may easily recognize the difference in buying from a direct and indirect e-stores (Mendez et al., 2008).

Third, a conceptual framework from a consumer perspective, antecedents and consequences of customer satisfaction, has been proposed and empirically proved. The literature (Alharthey, 2020) discussed different risk types in online shopping. Three main types of risk, perceived uncertainty, perceived risk, and price, are addressed in this model. To the best of the knowledge of the authors, no such investigation directed specific circumstances, particularly in the developing countries. Therefore, it is necessary to look for the antecedents and consequences of customer satisfaction to promote online shopping in the developing countries. The degree of consumer satisfaction defines his/her experience and emotions about the product or service purchased through the online channel. Recent studies (Guzel et al., 2020; Mamuaya and Pandowo, 2020) stated that the intention of the consumers to repurchase and their electronic-word-of-mouth (e-WOM) depends on their degree of satisfaction. In light of these heavy investments in online shopping, there is an exciting yet unexplored opportunity to comprehend better how the purchasing experiences of consumers through online channels influence their satisfaction level.

The study contributed to the current marketing literature in several ways. First, this study has highlighted that the perceived risk is very high when shopping through online channels, mainly the indirect e-stores. Therefore, the managers should develop strategies that reduce the perceived risk for the online consumer to shop more. Second, the study also disclosed that the perceived uncertainty in shopping through the online channel is high. While shopping online, the website design, graphics, and color scheme make the product more attractive than the actual one. Therefore, the managers must balance the visual appearance of the product on the website with the actual appearance of the product. This would increase the confidence and satisfaction of the consumer. Third, this study has also revealed that people are more satisfied while shopping from direct e-stores than indirect e-stores. Because the focal brands officially sponsor the direct e-stores, they pay more attention to their quality to retain consumers and maintain their brand reputation. Fourth, an indirect e-store works as a third party or a retailer who does not own the reputation of the product. This study exhibited the difference between the perception of the consumer being very high and the actual experience of using that product being quite different when shopping from the indirect channel. Last but not the least, this study is the first to report pre- and post-purchase consumer behavior and confirmed the perceived and the actual quality of a product bought from (i) direct e-store and (ii) indirect e-store.

## Literature Review

### Theoretical Review

Literature shows that when consumers get influenced to buy a particular product or service, some underlying roots are based on their behavior (Wai et al., 2019). Appraisal theory significantly explains consumer behavior toward shopping and provides an opportunity to analyze the evaluation process (e.g., Roseman, 2013; Kähr et al., 2016; Moors et al., 2017; Ul Haq and Bonn, 2018). This research, aligned with the four dimensions of appraisal theory as the first stage, clearly defines the agency stage that either of the factors is responsible for customer satisfaction. The second stage explains that consumer's degree of satisfaction holds great importance and refers to novelty in the literature. The third stage of the model briefly explains the feelings and emotions of the consumers about the incident, aligning with the certainty phase. The last step explains whether the consumers have achieved their goal or are not aligned with the appetitive purpose.

Cognitive appraisal researchers stated that various emotions could be its root cause (Scherer, 1997); it could be the reaction to any stimulus or unconscious response. On the contrary, four dimensions of appraisal theory are discussed in this research (Ellsworth and Smith, 1988; Ma et al., 2013). Agency (considering themselves or objects are answerable for the result of the circumstance) (Smith and Ellswoth, 1985; Durmaz et al., 2020); novelty (assessing the difference between the perception of an individual and his actual experience) (Ma et al., 2013); certainty (analysis of the apparent probability of a specific outcome and its effect on the emotions of the buyer) (Roseman, 1984), and appetitive goal (judging the degree to what extent the goal has been achieved) (Hosany, 2012).

### Hypotheses Development

#### Perceived Risk and Consumer Satisfaction

Perceived risk is the perception of shoppers having unpleasant results for buying any product or service (Gozukara et al., 2014). Consumers who buy a specific product or service strongly impact their degree of risk perception toward buying (Jain, 2021). Buyers who tend to indulge in buying through online channels face perceived risk characterized by their perception compared to the actual uncertainty involved in it (Kim et al., 2008). Literature (Ashoer and Said, 2016; Ishfaq et al., 2020) showed that as the risk of buying is getting higher, it influences the degree of consumers about information about their buying, either purchasing from the direct or indirect e-shop. Johnson et al. (2008) stated that consumer judgment that appears due to their experience strongly impacts their satisfaction level. Jin et al. (2016) said that as the ratio of risk perception of their consumer decreases, it enhances customer satisfaction. Thus, from the above arguments, it is hypothesized as follows:

**H**_**1**_: *Perceived risk has a significant negative impact on consumer satisfaction—direct* vs. *indirect e-store; perceived* vs. *actual experience*.

#### Perceived Uncertainty and Consumer Satisfaction

Uncertainty is defined as a time that occurs in the future that comprises the predictable situation due to the asymmetry nature of data (Salancik and Pfeffer, 1978). Consumers may not expect the outcome of any type of exchange conducted as far as the retailer and product-oriented elements are concerned (Pavlou et al., 2007). Therefore, uncertainty initiates that retailers may not be completely predictable; on the contrary, consumers tend to analyze and understand their actions about decision making (Tzeng et al., 2021). Thus, the degree of uncertainty involved in buying through online channels influences that degree of customer satisfaction. In addition, when the performance of any particular product or service matches the degree of expectations, he gets satisfied and, hence, repeats his decision of buying (Taylor and Baker, 1994). But if the product quality fails to meet the requirements, it negatively affects the degree of satisfaction (Cai and Chi, 2018).

**H**_**2**_: *Perceived uncertainty has a significant negative impact on consumer satisfaction—direct* vs. *indirect e-store; perceived* vs. *actual experience*.

#### Price Value and Consumer Satisfaction

Oliver and DeSarbo (1988) suggested that the price value is the proportion of the result of the buyer to the input of the retailer. It is defined as an exchange of products/services based on their quality against a price that is to be paid (Dodds et al., 1991). Consumers look for a higher value in return; consumers are willing to pay a higher price (Pandey et al., 2020). Yet, it leads to higher dissatisfaction when they receive a lower degree of profitable products. Besides, the buyers associate such type of product/service they use as less favorable or not according to their needs and desires. Hence, the buyers regret their decision-making degree for choosing that particular product (Zeelenberg and Pieters, 2007). Aslam et al. (2018) indicated that a product/service price influences the satisfaction of a buyer. Afzal et al. (2013) recommended that the price is among those factors that hold great significance for the degree of satisfaction of the consumer. If the price value of any product/service differs from consumer to consumer, consumers tend to switch brands. Hence, it is hypothesized that:

**H3**: *Price value has a significant positive impact on consumer satisfaction—direct* vs. *indirect e-store; perceived* vs. *actual experience*.

#### Consumer Satisfaction With Consumer Delight, Consumer Regret, and Outrage

Satisfaction is defined as how a consumer is pleased with a particular brand or view about a product/service that matches requirements. It is an essential factor that triggers when the product or service performance exceeds the expectation and perception of the customers (Woodside et al., 1989). The decision of the buyer significantly affects their satisfaction toward the product or service (Park et al., 2010). If buyers are satisfied with the product/service they purchased online, this degree of satisfaction significantly affects their repurchase intention and WOM (Butt et al., 2017). Tandon (2021) stated that a consumer satisfied with the product/service would get delighted. Consumer satisfaction, when exceeding the expectations, leads to consumer delight (Mikulić et al., 2021). Mattila and Ro (2008) recommended that the buyer gets disappointed by anger, regret, and outrage. It also defines that negative emotions have a significant effect on the purchasing intention of the consumers. Oliver (1989) stated that unsatisfied buyers or products that do not fulfill the needs of the customers can create negative emotions. Sometimes, their feelings get stronger and result in sadness and outrage. Bechwati and Xia (2003) recommended that the satisfaction of the consumers influences their behavior to repurchase; outraged consumers due to dissatisfaction sometimes want to hurt the company. Besides deciding to purchase, consumers mostly regret their choices compared to other existing choices (Rizal et al., 2018). Hechler and Kessler (2018) investigated that consumers who are outraged in nature actively want to hurt or harm the company or brand from which they got dissatisfied or hurt. Thus, it is proposed that:

**H**_**4**_: *Consumer satisfaction has a significant negative impact on (a) consumer delight, (b) consumer regret, (c) consumer outrage—direct* vs. *indirect e-store; perceived* vs. *actual experience*.

#### Consumer Delight and E-WOM

Oliver et al. (1997) recommended that a degree of delight in a buyer is termed as a positive emotion. Consumers purchase a product/service that raises their degree of expectation and gets them delighted (Crotts and Magnini, 2011). Delighted buyers are involved in sharing their experiences with their friends and family and spreading positive WOM to others (Parasuraman et al., 2020). Happy buyers generally share their opinions while posting positive feedback through social media platforms globally (Zhang, 2017). A positive WOM of the buyer acts as a fundamental factor in spreading awareness about the product/service and strongly impacts other buyers regarding buying it (Rahmadini and Halim, 2018). Thus, it is proposed that:

**H5**: *Consumer delight has a significant positive impact on E-WOM—direct* vs. *indirect e-store; perceived* vs. *actual experience*.

#### Consumer Delight and Repurchase Intention

Delighted consumers tend toward brand loyalty; thus, they increase their buying intention of the service or product (Ludwig et al., 2017; Ahmad et al., 2021). Customers can understand the objective of loyalty in purchasing a similar product or a new one from the same company. Delighted consumers tend to indulge in a higher degree of an emotional state that leads them to higher purchase intentions; it eliminates the switching of brands (Parasuraman et al., 2020). Kim et al. (2015) stated that consumers delighted with a product or service of a brand become loyal to it, and the possibility of switching brands gets very low. Research (Loureiro and Kastenholz, 2011; Tandon et al., 2020) shows that delighted consumers are more eager to purchase the same product again. Hence, it is proposed that:

**H6**: *Consumer delight has a significant positive impact on his repurchase intention—direct Vs. indirect e-store; Perceived Vs. actual experience*

#### Consumer Regret and E-WOM

Regret is considered a negative emotion in reaction to an earlier experience or action (Tsiros and Mittal, 2000; Kumar et al., 2020). Regret is when individuals frequently feel pity, disgrace, shame, or humiliation after acting in a particular manner and afterward try to amend their possible actions or decisions (Westbrook and Oliver, 1991; Tsiros and Mittal, 2000). Regret is that specific negative emotion the buyers feel while making a bad decision that hurts them; their confidence level is badly affected. They blame themselves for choosing or creating a terrible decision (Lee and Cotte, 2009). Li et al. (2010) suggested that buyers quickly start regretting and find their way to express their negative emotions. When they feel betrayed, they tend to spread negative WOM (NWOM) as a response to their anxiety or anger. Jalonen and Jussila (2016) suggested that buyers who get dissatisfied with their selections get involved in negative e-WOM about that particular brand/company. Earlier research says that buyers suffering from failure to buy any product/services tend to participate actively and play a role in spreading NWOM due to the degree of regret after making bad choices. Whelan and Dawar (2014) suggested that consumers sense that business has treated them unreasonably, and many consumers complain about their experience, resulting in e-WOM that may reduce consumer repurchase intention. Thus, it can be stated that:

**H7**: *Consumer regret has a significant negative impact on e-WOM—direct* vs. *indirect e-store; perceived* vs. *actual experience*.

#### Consumer Regret and Repurchase Intention

Regret has a substantial influence on the intentions of the consumers to not entirely be measured by their degree of happiness (Thibaut and Kelley, 2017). Results may not be evaluated by matching the structured degree of expectation but are also linked to alternatives reachable in the market. Therefore, such sort of evaluation and assessments will probably influence repurchase intention. For example, suppose the skipped reserve overtakes the picked alternative. In that case, the customer might change the replacement for the future purchase, regardless of whether the individual is profoundly happy with the picked option (Liao et al., 2017). According to the researchers, there is a negative relationship between regret and consumer repurchase intention (Liao et al., 2017; Durmaz et al., 2020). Furthermore, Unal and Aydin (2016) stated that perceived risk negatively impacts regret, influencing the repurchase intention of the consumers. Thus, it can be stated that:

**H8**: *Customer's regret has a significantly negative influence on his repurchase intention—direct* vs. *indirect e-store; perceived* vs. *actual experience*.

#### Consumer Outrage and E-WOM

The disappointment of the consumers is a negative response to a product or a service (Anderson and Sullivan, 1993). Outrage is the negative emotion a consumer experience when he purchases something totally against his requirements (Lindenmeier et al., 2012). Besides, when the perception of the buyer is infringed, such behaviors occur. According to Torres et al. (2019), enraged consumers get involved in communicating their outrage through e-WOM. Outraged consumers actively hurt the firm or brand from which they got hurt (Hechler and Kessler, 2018). Consumers give e-WOM online reviews to decrease the negative emotions from the experiences of the consumer and re-establish a calm mental state to equilibrium (Filieri et al., 2021). Thus, such consumers tend to give negative comments about the brand or product, which failed to match their expectations. NWOM has been characterized as negative reviews shared among people or a type of interpersonal communication among buyers concerning their experiences with a particular brand or service provider (Balaji et al., 2016). Hence, it is hypothesized that:

**H9**: *Consumer outrage has a significant negative impact on e-WOM—direct* vs. *indirect e-store; perceived* vs. *actual experience*.

#### Consumer Outrage and Repurchase Intentions

Repurchase intentions are characterized as the expressed trust of a buyer that they will or will not purchase a specific product and service again in the future (Malhotra et al., 2006). Establishing relations with buyers should result in the repurchase. Negative disconfirmation ensues dissatisfaction or a higher level of outrage (Escobar-Sierra et al., 2021). When a service/product fails and is not correctly addressed, the negative appraisal is overstated. Hence, “it may be more difficult to recover from feelings of victimization than to recover from irritation or annoyance” typically associated with dissatisfaction (Schneider and Bowen, 1999, p. 36). Therefore, consumers get outraged from buying such a product that fails to match their perception. When the experience of a consumer prompts a negative disconfirmation, the purchaser will also have a higher urging level through outrage. Therefore, consumers will probably have negative intentions to repurchase and do not want to indgule in making the same decision repeatedly (Wang and Mattila, 2011; Tarofder et al., 2016). Therefore, it is proposed that:

**H10**: *Consumer outrage has a significant negative impact on repurchase intention—direct* vs. *indirect e-store; perceived* vs. *actual experience*.

## Methodology

This research explores the difference between the perception of the consumers and the actual online shopping experience through direct and indirect e-stores. It was an experimental design in which online shopping was studied in the developing countries. Data were collected from those individuals who shop from online channels; direct e-store and indirect e-store. Taking care of COVID-19 standard operating procedures, only 50 respondents were gathered two times, every time in a university auditorium after obtaining the permission from the administration. The total capacity of the auditorium was 500, as the lockdown restrictions were lifted after the first wave of the coronavirus.

### Data Collection Tool

A questionnaire was used for the survey. The questionnaire was adapted in English to guarantee that the respondents quickly understood the questions used. A cross-sectional study technique was used for this research. A cross-sectional study helps in gathering the data immediately and collects data from a large sample size. The total number of distributed questionnaires was 1,250, out of which 800 were received in the usable form: 197 incomplete, 226 incorrect, and dubious responses, and 27 were eliminated. Thus, a 64% response rate was reported. Research showed that a 1:10 ratio is accepted (Hair et al., 1998) as far as the data collection is concerned; for that instance, this study data fell in the acceptable range.

#### Indirect E-Store

Consumers who prefer to shop through online channels were gathered in an auditorium of an institute. Only those consumers were eligible for this experiment, who themselves buy through e-stores. A few products were brought from an indirect e-store, and later on, those products were shown to the respondents from the website of that indirect e-store. After showing products, we asked the respondents to fill the survey as per their perception of the product. Then we asked them to fill out another questionnaire to ascertain the difference between the perception and actual experience when purchasing from an indirect e-store. Once all the respondents completed the survey, we have shown them the actual products they have selected by seeing the website of the indirect e-store.

#### Direct E-Store

The second experiment was carried out on those consumers who shop from direct e-stores. For that purpose, a few popular reviewed clothing articles were purchased from the e-store. As in the case of an indirect e-store, respondents were also shown these articles from the websites of these direct e-stores. We then asked the respondents to fill the survey to confirm their perception of the products. Once all the respondents completed the survey, we showed them the actual product and asked them to fill out another questionnaire according to their actual purchasing experience from the direct e-store. The primary purpose of this experiment was to compare buying from direct e-store and indirect e-store.

### Construct Instruments

The total number of items was 34, which were added in the earlier section of the questionnaire. These items were evaluated with the help of using a five-point Likert scale that falls from strongly disagree (1) to strongly agree (5). The items used in the study were empirically validated. **Table 2** carries the details of the items of the questionnaire. The price value was evaluated using three items used by Venkatesh et al. (2012). The perceived uncertainty was one of the independent variables that carry four items derived from Pavlou et al. (2007). Perceived risk was the third independent variable used, held three items; thus, its scale was derived from Shim et al. (2001). Wang (2011) validated consumer satisfaction carrying three items; consumer delight was measured by a 3-item scale proposed by Finn (2012); consumer regret was measured by the scale proposed by Wu and Wang (2017). It carries a three-item scale. Consumer outrage was measured by Liu et al. (2015); it has six items. Repurchase intention was measured through a scale adapted from Zeithaml et al. (1996), which carries four items. e-WOM was validated by the scale adapted from Goyette et al. (2010); it has five items.

## Results

### Demographics of the Respondents

**Figure 1:**
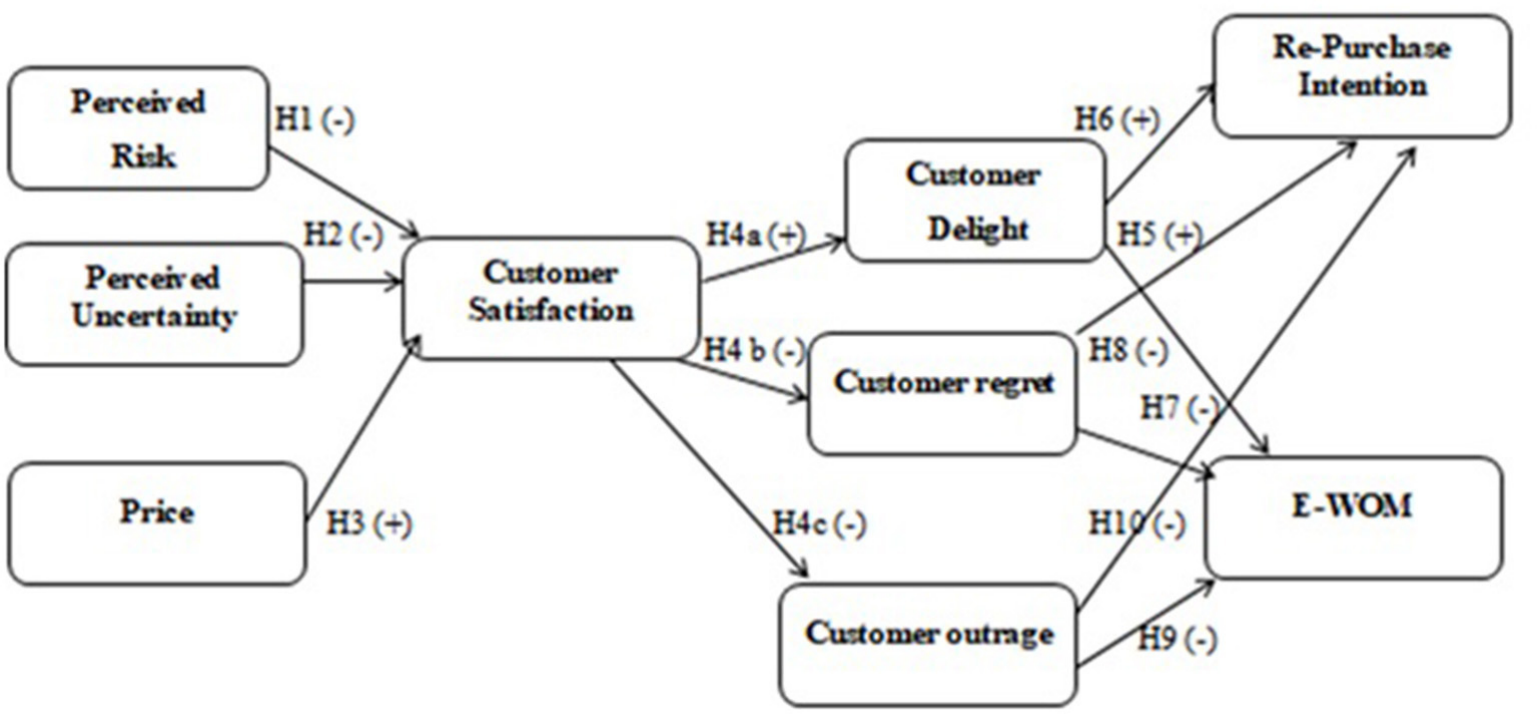
Proposed conceptual framework.

A total of 800 questionnaires were filled, and the respondents expressed their perception and actual experience from direct e-store and indirect e-store. Respondents belonged to different age groups from 18 to 50 years and above. There were 49% women and 51% men who took part in filling this survey. The income level of the respondents was grouped in different categories from “above 10,000 to above 50,000. The majority (56%) of the respondents were single, and 44% were married (Details can be viewed in Figure 1; Table 1). Data for both direct and indirect e-store was collected equally; 50% each to compare each category better.

**Table 1:** Demographics of the respondents.

**Demographics**		**Frequency**	**% of Total**
Age (years)	18–23	72	9
	24–30	248	31
	31–40	240	30
	41–50	192	24
	50 above	48	6
Gender	Male	392	49
	Female	408	51
Income level	>Rs 10,000	80	10
	Rs 10,000—Rs 20,000	272	34
	Rs 20,001—Rs 30,000	192	24
	Rs 30,001—Rs 40,000	112	14
	Rs 40,001—Rs 50,000	48	6
	Above 50,000	96	12
Marital status	Single	448	56
	Married	352	44
Direct	Direct	400	50
Indirect	Indirect	400	50
Visit	Everyday	80	10
	Weekly	360	45
	Monthly	333	42
	Once in several months	27	3
Purchase	Everyday	69	9
	Weekly	360	45
	Monthly	346	43
	Once in several months	25	3
Indirect stores	Amazon	56	7
	Daraz	144	18
	Ali Express	208	26
	11^th^ Street	200	25
	Other	192	24
Direct stores	Khaadi	280	35
	Nishat Linen	174	22
	Outfitters	40	5
	Al-Karam	152	19
	Others	150	19

### Reliability and Validity

Reliability evaluates with the help of composite reliability (CR). All CR values fall into the range of 0.7–0.9, which is acceptable (Hair et al., 2011). Convergent and discriminant validity has been observed through confirmatory factor analysis as recommended by some researchers (Fornell and Larcker, 1981; Hair et al., 2010).

#### Convergent Validity

Convergent validity is evaluated with the help of two standards mentioned in the literature earlier, factor loading and average variance extracted (AVE), both the values should be >0.5 (Yap and Khong, 2006). The values are mentioned in Table 2.

**Table 2:** Reliability and convergent validity.

**Variables**	**Items No**	**Items**	**FL**	**CR**	**AVE**
Price value	P1	Products purchase from the online shop is reasonably priced.	0.374	0.709	0.549
	P2	Internet shopping is a good value for the money	0.138		
	P3	At the current price, online shopping provides a good value	0.697		
Perceived Uncertainty	PUN1	I feel that purchasing through an online channel involves a high degree of uncertainty	0.823	0.907	0.715
	*PUN2*	I feel the uncertainty associated with online shopping is high	0.939		
	*PUN 3*	I am exposed to many transaction uncertainties if I fill in my details while shopping through an online channel	0.961		
	*PUN 4*	There is a high degree of product uncertainty (i.e., the product you receive may not be exactly what you want) when purchasing through an online store	0.615		
Perceived risk	*PR1*	Shopping on the Internet is risky	0.969	0.913	0.778
	*PR2*	There is too much uncertainty associated with shopping on the Internet	0.827		
	*PR 3*	Compared with other methods of purchasing, Internet shopping is riskier.	0.843		
Customer satisfaction	*CS 1*	I feel comfortable with shopping from here	0.847	0.947	0.857
	*CS 2*	The product or service was satisfying to me	0.940		
	*CS 3*	The service or product which I got was worth the time I spent on it.	0.978		
Consumer delight	*CD 1*	I was delighted by the visit	0.904	0.926	0.661
	*CD2*	I (will) happily talk about the visit.	0.892		
	*CD3*	I was overjoyed with the visit	0.893		
Consumer regret	CR1	I regret buying the product	0.983	0.926	0.807
	CR2	I should not have chosen the product	0.824		
	CR3	I feel sorry for buying the product	0.880		
Consumer outrage	OUT1	They made me so angry	Del	0.886	0.661
	OUT 2	I left the product in a rage	del		
	OUT 3	I never thought that I could feel so mad toward	0.848		
	OUT 4	I cannot believe that I could hate a restaurant/e-store so much	0.787		
	OUT 5	I felt like beating someone after shopping	0.861		
	OUT 6	I was very outraged by the product	0.754		
Repurchase intention	*PUR 1*	I plan to keep on buying this same product and brand in the future.	0.999	0.891	0.677
	*PUR 2*	I will consider this brand as my first option to the purchase of other products	0.713		
	*PUR 3*	In the future, if I purchase a new product, I will privilege this brand over the competitor (alternative brands	0.679		
	*PUR 4*	I intend to buy products of this same brand more frequently in the future.	0.861		
e-WOM	*e-WOM 1*	I often read online recommendations to buy products through online channels.	0.880	0.935	0.781
	*e-WOM 2*	I often post online comments about online retailers	0.930		
	*e-WOM 3*	I often read online reviews about the products of online retailers	Del		
	*e-WOM4*	My e-community frequently post online recommendations to buy from online retailers	0.874		
	*e-WOM 5*	When I buy a product from online retailers, online recommendations and reviews of consumers make me more confident in purchasing the product	0.847		

#### Discriminant Validity

Discriminant validity is evaluated based on two conditions that are required to evaluate it. First, the correlation between the conceptual model variables should be <0.85 (Kline, 2005). Second, the AVE square value must be less than the value of the conceptual model (Fornell and Larcker, 1981). Table 3 depicts the discriminant validity of the construct of the study.

**Table 3:** Discriminant validity.

	**OUT**	**P**	**CS**	**PUN**	**CR**	**e-WOM**	**PR**	**CD**	**Repur**
Outrage	**0.718**								
Price	−0.031	**0.837**							
Customer satisfaction	−0.026	0.705	**0.929**						
Uncertainty	−0.017	−0.067	−0.185	**0.845**					
Regret	0.024	0.304	0.268	−0.066	**0.884**				
Word of mouth	0.212	0.026	0.079	−0.311	0.234	**0.880**			
Risk	−0.022	0.099	0.236	−0.297	0.055	0.189	**0.882**		
Delight	0.082	0.265	0.337	−0.149	0.148	0.190	0.266	**0.873**	
Repurchase	−0.056	−0.021	−0.017	0.039	0.169	0.021	0.008	0.196	**0.807**

*All diagonal bold values are square root of AVE*.

### Multi-Group Invariance Tests

Multi-group confirmatory factor analysis was conducted as the pre-requisites for the measurement model. The multi-group analysis was used to investigate a variety of invariance tests. Different invariance tests were performed to guarantee the items working precisely in the same manner in all the groups. In this research, the following are the model fit indexes, that is, CMIN/dF =2.992 CFI = 0.915, TLI = 0.906, and RMSEA = 0.071. Byrne (2010) and Teo et al. (2009) stated that CFI gives more accurate results, especially when comparing variables in different groups.

### Hypotheses Testing

Scanning electron microscope technique was used to run and test the proposed hypotheses for the conceptual model. First, all the hypotheses proposed were checked, from which eight were initially accepted. Later, the multi-group test was utilized to test the proposed hypotheses and compare the shopping experience from direct e-store with indirect e-store and consumer perception with actual experience. Table 4 explains this in detail.

**Table 4:** Hypotheses results.

	**Hypotheses**	**Group**	**Standardized estimates**	**Supported/** **not supported**
H1a	PR → CS	Direct	0.6^**^	Supported
H1 b		Indirect	0.011^**^	Supported
H1 c		Perceived	0.032^**^	Supported
H1 d		Actual	0.026^**^	Supported
H2	PUN → CS			
H2 a		Direct	0.40^*^	Supported
H2 b		Indirect	0.018^*^	Supported
H2 c		Perceived	0.018^**^	Supported
H2 d		Actual	0.031^*^	Supported
H3	P → CS			
H3a		Direct	0.191^*^	Supported
H3 b		Indirect	0.397^*^	Supported
H3 c		Perceived	0.524^*^	Supported
H3 d		Actual	0.399^*^	Supported
H4 (i)	CS → CD			
H4a		Direct	0.115^*^	Supported
H4 b		Indirect	0.051^**^	Supported
H4 c		Perceived	0.051^*^	Supported
H4 d		Actual	0.061^*^	Supported
H4 (ii)	CS → CR			
H4a		Direct	−0.0115	Supported
H4 b		Indirect	−0.051	Supported
H4 c		Perceived	−0.061	Supported
H4 d		Actual	−0.070	Supported
H4 (iii)	CS → OUT			
H4a		Direct	−0.093	Not Supported
H4 b		Indirect	−0.016	Supported
H4 c		Perceived	−0.052	Supported
H4 d		Actual	−0.025	Supported
H5	CD → E-WOM			
H5a		Direct	0.056	Supported
H5 b		Indirect	0.063	Not Supported
H5 c		Perceived	0.053	Supported
H5 d		Actual	0.053	Supported
H6	CD → PUR			
H6a		Direct	0.55	Supported
H6b		Indirect	0.060	Not Supported
H6 c		Perceived	0.052	Supported
H6 d		Actual	0.051	Supported
H7	CR → E-WOM			
H7a		Direct	−0.47	Supported
H7 b		Indirect	0.045	Not Supported
H7 c		Perceived	−0.050	Supported
H7 d		Actual	−0.044	Supported
H8	CR → PUR			
H8a		Direct	−0.045	Supported
H8 b		Indirect	−0.043	Supported
H8 c		Perceived	−0.050	Supported
H8 d		Actual	−0.044	Supported
H9	OUT → E-WOM			
H9a		Direct	−0.059	Supported
H9 b		Indirect	−0.193	Supported
H9 c		Perceived	−0.062	Supported
H9 d		Actual	−0.140	Supported
H10	OUT → PUR			
H10a		Direct	0.055	Not Supported
H10 b		Indirect	0.146	Not Supported
H10 c		Perceived	0.061	Not Supported
H10 d		Actual	0.116	Not Supported

** *P < 0.001*,

* *P < 0.01*.

## Discussion and Implications

This research offers a remarkable number of facts for practitioners. This study can benefit marketing strategists by reducing the perceived risk, decreasing the intensity of perceived uncertainty, stabilizing the price, enhancing consumer satisfaction, promoting delighting consumers, accepting the negative behavior of the consumers, consumer retention, and establishing a positive e-WOM.

### Reducing Risks

Certain factors play a role in antecedents of consumer satisfaction; they are particularly those that resist consumers to shop from any online channel, neither direct e-store nor indirect e-store. Perceived risk, perceived uncertainty, and the price are some of those antecedents that play a significant role in affecting the degree of satisfaction of the consumers, resulting in either to retain a consumer or to outrage a consumer. This study aligns with the existing literature. Tandon et al. (2016); Bonnin (2020) and Pandey et al. (2020) showed that consumers seek to shop from an e-store without bearing any risk. Consumers feel more confident about an e-store when the perceived risk is less than shopping from traditional ones as consumers want to feel optimistic about their decision. Second, e-vendors should ensure that the quality of a product is up to the mark and according to the consumer needs. Therefore, vendors should offer complete details about the product/service and its risks to the consumers. Moreover, this study suggests that e-stores must align the visuals of a product with its actual appearance. This would help them to increase customer satisfaction and confidence in the e-store.

### Focus on Consumer Satisfaction

Consumer satisfaction is the deal-breaker factor in the online sector. Literature (Shamsudin et al., 2018; Hassan et al., 2019) showed that organizations prioritize their consumers by fulfilling their requirements and required assistance. As a result, consumers are more confident and become satisfied consumers in the long run. This study adds to the literature that the degree of satisfaction of the consumers plays an essential role in shopping from an e-store. Consumers feel more confident in shopping from a direct e-store than an indirect e-store as the difference in the perception of consumers and the actual experience varies. Therefore, online vendors should focus on satisfying their consumers as it plays a remarkable role in retaining consumers.

### Value Consumer Emotions

Online, retaining, and satisfying consumers are the most vital factor that directly affects the organization. This research aligns with the existing literature (Jalonen and Jussila, 2016; Hechler and Kessler, 2018; Coetzee and Coetzee, 2019); when the retailer successfully fulfills its requirements, the consumer gets delighted repeating his choice to repurchase. On the other hand, if the online retailer fails to serve the consumer, the consumer regrets and, in extreme cases, becomes outraged about his decision. The negative emotions of the consumers threaten the company from many perspectives, as the company loses its consumer and its reputation in the market is affected. Therefore, first, market practitioners should avoid ignoring the requirements of consumers. Second, online vendors should pay special attention to the feedback of the consumers and assure them that they are valued.

### Consumer Retention

The ultimate goal is to retain its consumers, but e-vendors should make proper strategies to satisfy their consumers as far as the online sector is concerned. The earlier studies of Zhang et al. (2015) and Ariffin et al. (2016) contributed to the literature that consumer satisfaction is a significant aspect in retaining a consumer. This research has also suggested that the satisfaction of the consumers plays a vital role in retaining them. Moreover, online shoppers provide the fastest spread of the right WOM about the product/ service. Second, consumers should feel valued and committed to vendors.

### Pre- and Post-buying Behavior

This study contributed to a conceptual model that deals with consumer pre- and post-purchase behavior from the direct and indirect e-stores. With the help of experimental design, this study has reported its finding, highlighted how a satisfied customer is delightful and shares e-WOM, and showed repurchase intention. However, if the customer is not satisfied with the flip of a coin, he may feel regretted or outraged and cannot share e-WOM or have a repurchase intention.

## Conclusions

This research concludes that online shopping has boomed during this COVID-19 pandemic period, as the lockdown prolonged in both the developed and the developing countries. The study further supports the difference between shopping from a direct e-store and an indirect e-store. The perception of the consumers shopping from direct e-store is more confident, and their degree of satisfaction is much higher, as the actual experience of the consumers aligns with their perceptions. Instead, consumers feel dissatisfied or outraged to choose an indirect e-store for shopping. Indirect e-store makes false promises and guarantees to its buyers, and eventually, when the consumers experience the product, it is against their perception.

This research fills the literature gap about the antecedents that lead to online shopping growth in the developing countries. This study aligns with Hechler and Kessler's (2018) earlier research, which stated that dissatisfied consumers threaten the reputation of the organization. Furthermore, Klaus and Maklan (2013), Lemon and Verhoef (2016) suggested that handling the experience and satisfaction of the buyers plays a significant role in surviving among its competitors. Grange et al. (2019) recommended that e-commerce develops and attracts consumers by fulfilling their needs and requirements quickly. This study aligned with the existing literature by adding factors influencing the shopping preferences of the consumers from an e-store.

### Limitations and Future Research

Despite its significant findings, this research has some limitations and scope for future research. First, this research only examined a few risks involved in online shopping. Future research studies should analyze other risks, for example, quality risk and privacy risk. Second, this study focused on shopping through direct e-stores and indirect e-stores. Future research can implement a conceptual model of a specific brand. Third, this study can be implemented in other sectors, for example, tourism, and hospitality. Fourth, it may be fascinating to look at other fundamentals, such as age, gender, education, relation with the retailer, or the degree of involvement with online shopping to differentiate other factors.

The proposed framework can be utilized in other developing countries, as every country faces different problems according to its growth and development. The model can be examined among specific direct e-stores to compare new customers and loyal customers. Future studies can explore indirect relationships along with adding mediators and moderators in the proposed model.

## Data Availability Statement

The original contributions presented in the study are included in the article, further inquiries can be directed to the corresponding author.

## Ethics Statement

The studies involving human participants were reviewed and approved by This study involving human participants was reviewed and approved by the Ethics Committee of the Department of Management Sciences, Riphah International University, Faisalabad Campus, Faisalabad, Pakistan. The participants provided their written informed consent to participate in this study. The patients/participants provided their written informed consent to participate in this study.

## Author Contributions

AS contributed to the conceptualization and writing the first draft of the research. JU contributed to visualizing and supervising the research. All authors who contributed to the manuscript read and approved the submitted version.

## Conflict of Interest

The authors declare that the research was conducted in the absence of any commercial or financial relationships that could be construed as a potential conflict of interest.

## Publisher's Note

All claims expressed in this article are solely those of the authors and do not necessarily represent those of their affiliated organizations, or those of the publisher, the editors and the reviewers. Any product that may be evaluated in this article, or claim that may be made by its manufacturer, is not guaranteed or endorsed by the publisher.
